# The proliferative human monocyte subpopulation contains osteoclast precursors

**DOI:** 10.1186/ar2616

**Published:** 2009-02-17

**Authors:** Roya Lari, Peter D Kitchener, John A Hamilton

**Affiliations:** 1 Department of Medicine and Cooperative Research Centre for Chronic Inflammatory Diseases, University of Melbourne, The Royal Melbourne Hospital, Parkville, Victoria 3050, Australia; 2 Department of Anatomy and Cell Biology, University of Melbourne, Parkville, Victoria 3010, Australia

## Abstract

**Introduction:**

Immediate precursors of bone-resorbing osteoclasts are cells of the monocyte/macrophage lineage. Particularly during clinical conditions showing bone loss, it would appear that osteoclast precursors are mobilized from bone marrow into the circulation prior to entering tissues undergoing such loss. The observed heterogeneity of peripheral blood monocytes has led to the notion that different monocyte subpopulations may have special or restricted functions, including as osteoclast precursors.

**Methods:**

Human peripheral blood monocytes were sorted based upon their degree of proliferation and cultured in macrophage colony-stimulating factor (M-CSF or CSF-1) and receptor activator of nuclear factor-kappa-B ligand (RANKL).

**Results:**

The monocyte subpopulation that is capable of proliferation gave rise to significantly more multinucleated, bone-resorbing osteoclasts than the bulk of the monocytes.

**Conclusions:**

Human peripheral blood osteoclast precursors reside in the proliferative monocyte subpopulation.

## Introduction

Rheumatoid arthritis (RA) is a chronic disease that is characterized by joint inflammation and profound focal and generalized bone loss due to the action of osteoclasts [1,2]. Multinucleated osteoclasts derive from monocyte/macrophage lineage precursors; two key mediators controlling their development are macrophage colony-stimulating factor (M-CSF or CSF-1) and receptor activator of nuclear factor-kappa-B ligand (RANKL) [3-5]. Human osteoclast precursors have been shown to be present at low frequency in normal peripheral blood [6-8]. It now appears that peripheral blood monocytes, which derive from bone marrow precursors, are heterogeneous as judged by criteria such as surface marker expression, size, and function [9]. For example, in the human, there is a minor subpopulation of monocytes which is CD14^lo ^CD16^+ ^[10] and which has been implicated in inflammation and cancer [11-13]; in the mouse, there is a lot of recent interest in monocyte subpopulations that appear to have different roles during inflammatory reactions as manifested, for example, by their ability to migrate to sites of inflammation [14]. Human osteoclast precursors have recently been shown to reside in the CD14^+ ^CD16^- ^monocyte subpopulation of normal donors [15]. Blood samples from psoriatic arthritis patients, particularly those with bone erosions visible on plain radiographs, exhibit an increase in osteoclast precursors compared with those from healthy controls [16]; these precursors were recently reported to reside in the CD14^lo ^CD16^+ ^monocyte subset, leading the authors to suggest that osteoclasts are derived from distinct monocyte subsets in these patients and in healthy individuals [17].

Human monocytes are commonly considered to be non-proliferating [18]; however, we and others have defined a subpopulation of human monocytes which is capable of proliferating *in vitro *(for example, in response to M-CSF) [19-25]. This population has been referred to as proliferative monocytes (PMs), which were shown recently to have the phenotype CD14^+^ CD16^- ^CD64^+ ^CD33^+ ^CD13^lo ^c-Fms^+ ^prior to culture [25]. It was previously suggested that PMs might be able to migrate into inflamed tissues and possibly undergo local proliferation there [19,25]. During these prior phenotyping studies, it was noticed in passing that, following culture and sorting by flow cytometry, the PMs, from the few donors studied, could give rise to tartrate-resistant acid phosphatase-positive (TRAP^+^) multinucleated cells upon culture in M-CSF + RANKL [25]. Based on this preliminary observation and the likelihood that the PMs represent a relatively less mature monocyte population on account of their ability to proliferate, it was reasoned that they may retain differentiation capability and therefore contain the osteoclast precursors. We present evidence here for this concept for the peripheral blood from normal donors.

## Materials and methods

### Peripheral blood mononuclear cell isolation, CFSE labeling, and cell culture

Peripheral blood mononuclear cells (PBMCs) were isolated following Ficoll centrifugation and labeled with carboxyfluorescein diacetate-succinimidyl ester (CFSE) (Molecular Probes Inc., now part of Invitrogen Corporation, Carlsbad, CA, USA) as described previously [25]. CFSE-labeled PBMCs were seeded onto non-treated 100-mm dishes (Iwaki; Asahi Techno Glass Corporation, Funabasi City, Japan) at a concentration of 3 to 5 × 10^7 ^cells per dish and allowed to adhere overnight in alpha-minimum essential medium (α-MEM) (JRH Biosciences, now part of SAFC Biosciences, Lenexa, KS, USA) containing L-glutamine (2 mM; Invitrogen Corporation) and penicillin (100 U/mL)/streptomycin (100 μg/mL) (Invitrogen Corporation). Non-adherent cells were washed away, and new medium was added (α-MEM containing 3% heat-inactivated fetal bovine serum [Hi-FBS]) (CSL, Parkville, Victoria, Australia) with M-CSF (8,000 U/mL) (Chiron, Emeryville, CA, USA). These cultures were incubated at 37°C in 5% CO_2 _for 9 days with a change of medium and removal of non-adherent cells every 3 days.

### Cell sorting

The CFSE-stained cells were incubated in ice-cold phosphate-buffered saline (PBS) for 30 minutes and harvested by gentle scraping with a rubber policeman. Cells were resuspended in fluorescence-activated cell sorting (FACS) buffer (PBS containing 1% FBS) (Invitrogen Corporation) and 1 mM ethylenediaminetetraacetic acid (EDTA) (Ajax Chemicals, Cheltenham, Victoria, Australia) at a density of 10^7 ^cells per millilitre. Propidium iodide solution (3 μL of 1 mg/mL; Sigma-Aldrich, St. Louis, MO, USA) was added immediately prior to sorting. CFSE fluorescence levels were determined by flow cytometry. The appearance of a peak with high fluorescence intensity (CFSE^hi^) indicated the cells that had not divided. Half the fluorescence intensity (CFSE^lo^) indicated cells that have undergone one division. The existence of multiple peaks in some samples indicated multiple cell divisions in those populations [25]. CFSE-labeled cells were then sorted using a FACSVantage SE (BD Biosciences, San Jose, CA, USA).

### Osteoclast generation from peripheral blood mononuclear cells

Sorted cells were cultured at 3 × 10^4 ^cells per well (in α-MEM and 3% Hi-FBS) in M-CSF (8,000 U/mL; Chiron) with or without RANKL (50 ng/mL; PeproTech, Rocky Hill, NJ, USA). These cultures were incubated at 37°C in 5% CO_2 _for up to 21 days; the culture medium, including the relevant mediators, was changed twice per week. For the bone resorption assay, bone slices (horse cortical femur) were added to the well prior to the addition of the cells.

### Tartrate-resistant acid phosphatase staining

Osteoclast differentiation was determined firstly by TRAP staining following fixation in formaldehyde and acetone/alcohol as described previously [26]. Briefly, following fixation, cells were stained with freshly prepared TRAP staining solution (naphthol AS-MX phosphate, fast red violet LB salt, and potassium sodium tartrate). Osteoclast formation was evaluated by counting the TRAP^+ ^multinucleated (n ≥ 3) cells.

### mRNA extraction and quantitative reverse transcription-polymerase chain reaction analyses

Cells were plated at a density of 5 × 10^5 ^in 3 mL/well of medium (α-MEM and 3% Hi-FBS) in the presence of M-CSF (8,000 U/mL) with or without RANKL (50 ng/mL) in 6-cm tissue culture dishes (Becton, Dickinson and Company, Franklin Lakes, NJ, USA). Cells were incubated for 14 days with a complete change of medium every 3 to 4 days. Total RNA was isolated with the RNAeasy kit (Qiagen Inc., Valencia, CA, USA) in accordance with the instructions of the manufacturer. cDNAs were synthesized as described previously [27]. Pre-Developed TaqMan Assay Reagents (Applied Biosystems, Scoresby, Victoria, Australia) were used for cDNA sequence analysis for calcitonin receptor (CTR) and cathepsin K (Cath K). Quantitative polymerase chain reaction (PCR) analyses were used to quantify transcripts with the ABI Prism 7900 HT Sequence Detection System (Applied Biosystems) as described previously [27]. For the PCR analyses, fluorescence from each sample was measured once each cycle during PCR and plotted against cycle number; the earlier a signal appeared (at a lower cycle number), the higher the concentration of the template. The cycle threshold (Ct) number was used to indicate gene expression.

### Pit formation assay

Cells were removed from bone slices by brief sonication (approximately 30 seconds) and lysed in 1% Triton-X 100 for 30 minutes. Haematoxylin was applied to the resorbed surface of each slice for 1 minute and then the slices were washed three or four times with tap water. The residual stain was removed by wiping against absorbent paper. Resorption was observed by transmission light microscopy. Total pit area and total bone area were measured in 10 randomly selected areas for two or three bone slices by the Scion Image analysis program (Scion Corporation, Frederick, MD, USA), and the percentage pit area in each group was calculated [28].

### Statistical analysis

Data are presented as mean ± standard error. Significant differences were determined using the paired Student *t *test; a *P *value of less than or equal to 0.05 was considered significant.

## Results

### Proliferative monocytes contain precursors of tartrate-resistant acid phosphatase-positive multinucleated cells

**Figure 1:**
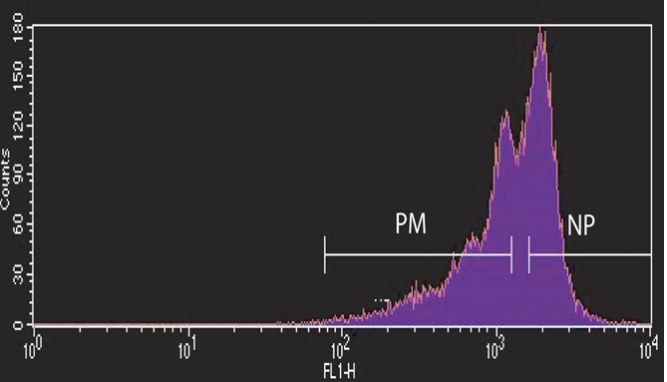
Sorting proliferative monocyte (PM) and non-proliferative (NP) population cells after carboxyfluorescein diacetate-succinimidyl ester (CFSE) labeling and culture. CFSE-labeled peripheral blood mononuclear cells were cultured in alpha-minimum essential medium + 3% heat-inactivated fetal bovine serum containing macrophage colony-stimulating factor (8,000 U/mL) in non-treated dishes for 9 days. The adherent cells were then sorted based on their CFSE fluorescence intensity as PM (CFSE^lo^) and NP (CFSE^hi^) populations [25].

**Figure 2:**
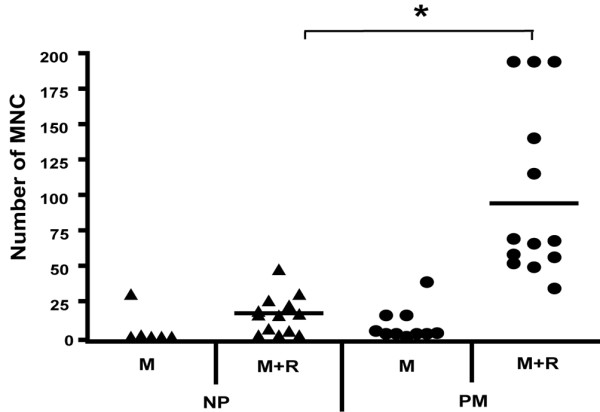
Proliferative monocytes (PMs) contain precursors of tartrate-resistant acid phosphatase-positive (TRAP^+^) multinucleated cells (MNCs). Non-proliferative (NP) and PM subpopulations from 13 donors, sorted as in Figure 1, were cultured in duplicate or triplicate cultures in macrophage colony-stimulating factor (M-CSF) (8,000 U/mL) and receptor activator of nuclear factor-kappa-B ligand (RANKL) (50 ng/mL) for 21 days; because insufficient cells were available, the two starting populations from fewer donors were also cultured in M-CSF alone. TRAP^+ ^MNCs were counted. The mean number of such cells was significantly higher in the PM-derived cells cultured in M-CSF and RANKL compared with the NP-derived population (*P < 0.001). M, macrophage colony-stimulating factor; R, receptor activator of nuclear factor-kappa-B ligand.

After culture in M-CSF, adherent, CFSE-labeled PBMCs could be sorted, based on their differing fluorescence intensities due to the number of cell divisions, into the PM and non-proliferative (NP) populations [25] (Figure 1). Preliminary data using PBMCs from three donors indicated that the former, predominantly spindle-shaped, population contained the bulk of the precursors which could be converted by culture in M-CSF + RANKL into TRAP^+ ^multinucleated cells with more intense TRAP staining (that is, possibly osteoclasts) [25]. In a more complete study, we now present data (Figure 2) for the number of TRAP^+ ^multinucleated (n ≥ 3) cells obtained from PBMCs from 13 donors and it can be seen that in general there were more of such cells derived from the PM population (*P *< 0.001) than from the NP monocyte population, an effect requiring the presence of RANKL; some multinucleated (n ≥ 3) cells could be observed even at day 7 in the PM cultures in the presence of M-CSF and RANKL (data not shown).

### Higher osteoclast-associated gene expression in the proliferative monocyte population following culture in M-CSF and RANKL

**Figure 3:**
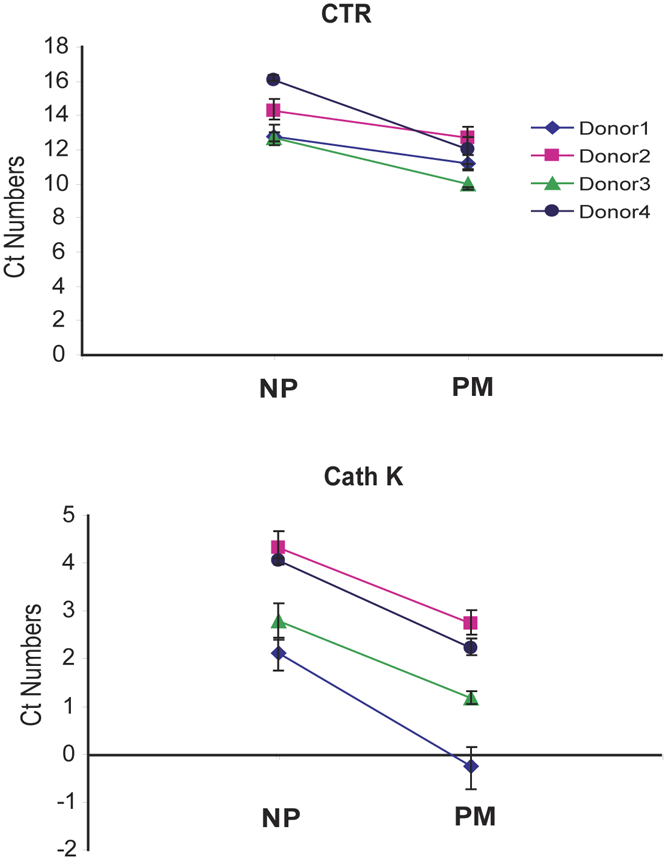
Osteoclast gene expression in differentiated proliferative monocyte (PM) and non-proliferative (NP) subpopulations. NP and PM subpopulations, sorted as in Figure 1, were cultured for 14 days in macrophage colony-stimulating factor (8,000 U/mL) and receptor activator of nuclear factor-kappa-B ligand (50 ng/mL). Calcitonin receptor (CTR) and cathepsin K (Cath K) mRNA expression were measured by quantitative polymerase chain reaction. Samples from four individual donors were tested in triplicate, and data were normalized to 18S expression for each gene. Values are means of cycle threshold (Ct) numbers that were obtained in each sample ± standard error. The mean values for the PM population were significantly lower than those for the correspondingly treated NP population from the same donor (*P *≤ 0.05).

Even though it was presented above that significantly more TRAP^+ ^multinucleated cells can be obtained from the PM population, actual osteoclast differentiation needs to be confirmed as osteoclasts and macrophage polykaryons are morphologically similar [29]; in addition, TRAP staining does not distinguish very well between such populations in the human. We therefore firstly measured the expression of certain genes whose products are associated with osteoclast function. In Figure 3, the results from four donors for CTR and Cath K mRNA expression following culture in M-CSF + RANKL for 14 days are provided; it can be noted that there was significantly more expression of these osteoclast-specific genes from the PM population, which required RANKL to be present (data not shown). Consistent again with the greater osteoclastogenic potential of the PMs, their progeny, following culture in M-CSF + RANKL, had significantly greater RANK expression when measured at 14 days, at least at the gene level, when compared with that from the NP cells (data not shown).

### Higher bone resorption in the proliferative monocyte population following culture in M-CSF and RANKL

**Figure 4:**
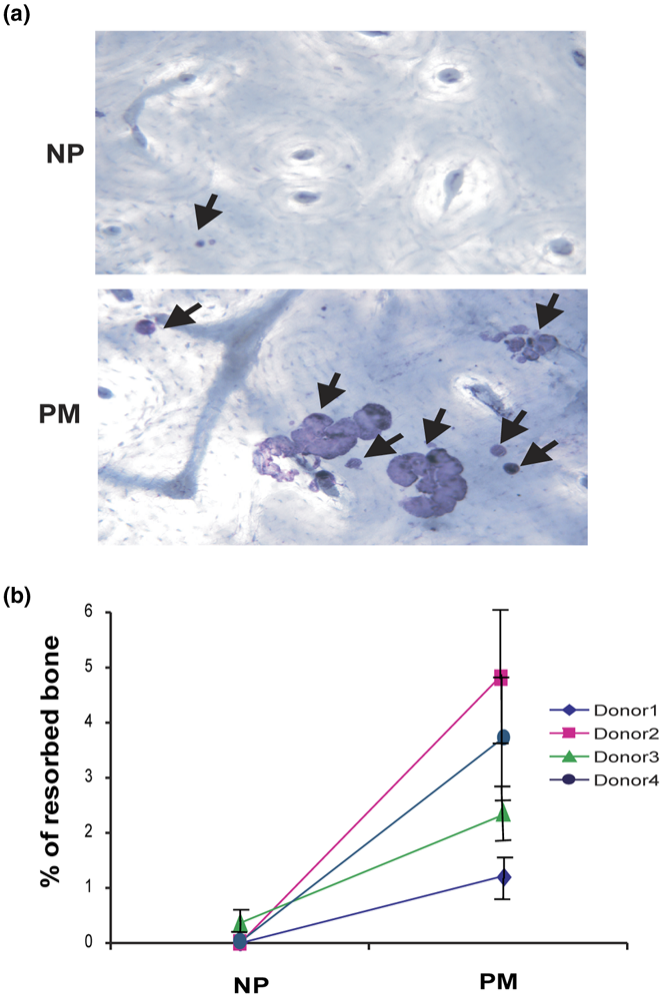
Precursors of bone-resorbing cells reside in the proliferative monocyte (PM) population. Sorted non-proliferative (NP) and PM populations (Figure 1) were cultured on bovine bone (3 × 10^4 ^cells per slice) for 21 days in the presence of macrophage colony-stimulating factor (8,000 U/mL) and receptor activator of nuclear factor-kappa-B ligand (50 ng/mL). **(a)** The bone slices were stained with haematoxylin (magnification × 200). Arrows indicate pits on the bone surface. **(b)** Resorption pit area measured for four donors (see 'Higher bone resorption in the proliferative monocyte population following culture in M-CSF and RANKL' section). Values are means of percentage of resorbed bone ± standard error. For each donor, the mean values for the PM group are significantly greater than those for the correspondingly treated NP cells (*P *< 0.05).

To confirm the functional activity of the multinucleated cells produced, bone resorption was measured next. The PM and NP populations were cultured in tissue culture dishes containing bone slices in the presence of M-CSF + RANKL. After 3 weeks, numerous resorption lacunae were found distributed over the surface of the bone slices in the PM cultures. However, only a few small resorption pits were observed in the bone slices cultured with NP cells under the same conditions (Figure 4).

## Discussion

Under steady-state conditions, osteoclastogenesis and bone remodeling occur mainly in the bone marrow. Osteoclast precursors can be mobilized from bone marrow into blood and then into tissues, particularly in some conditions involving bone loss at diseased sites (for example, RA) [30,31]. At an early stage of differentiation, they are also able to give rise to different myeloid populations [32], whereas at a later stage their differentiation involves M-CSF-dependent action on c-Fms^+ ^populations [33].

The heterogeneity of peripheral blood monocytes has led to the concept that there may be distinct subpopulations of cells with specialized functions [10,14,34,35]. For example, for the human, it is known that only a small proportion of monocytes can differentiate into osteoclasts [36,37]. Likewise, it is known that a small proportion of CD14^+ ^human monocytes (that is, PMs) can proliferate *in vitro *[19,25]; because of their ability to proliferate, it was reasoned that this less mature population, possibly representing cells recently mobilized from bone marrow, may be able to differentiate into different macrophage lineage populations, such as osteoclasts, under appropriate conditions.

Taking advantage of the relative ability of monocyte populations to undergo proliferation, we were able to show above that, for the blood from 13 donors, osteoclast precursors reside predominantly in the PM population and could be detected even after proliferation in M-CSF. Following further culture in M-CSF and RANKL, the resultant population containing the multinucleated progeny showed increased expression of certain osteoclast markers (CTR, Cath K, and RANK) and an ability to resorb bone. These findings are consistent with the concept that the PMs represent a less mature population, when compared with the bulk of the human peripheral blood monocytes [19-21], with some cells in the PM fraction at least retaining an ability to differentiate into osteoclasts. The data presented are consistent with prior observations that both the PM population [19,25] and osteoclast precursors [15] from normal individuals reside in the CD14^+ ^CD16^- ^monocytes rather than in the CD14^lo ^CD16^+ ^population, that osteoclastic cells can be generated from proliferating dendritic cell precursors in human peripheral blood [38], and that there is an early increase in the percentage of human peripheral blood osteoclast precursors entering S phase during their *in vitro *differentiation in M-CSF + RANKL [39]. It is possible that the PMs can differentiate while in the blood into NP monocytes with reduced proliferative and differentiation potential, perhaps under the influence of circulating M-CSF.

It is intriguing that, in psoriatic arthritis, the opposite finding has been made in that the increased numbers of peripheral blood osteoclast precursors noted were located in the CD16^+ ^population [17]. It would be worth knowing whether the PM population also increases in this and perhaps other inflammatory conditions and whether they begin to express higher CD16 levels *in vivo*. We suggest again [25] that functional criteria, such as PM status, have an advantage over surface marker phenotyping in that they avoid the difficulty in defining, for example, for monocyte populations whether modulation in the expression of a particular surface marker reflects differentiation or activation.

## Conclusion

In summary, it has been shown here that human peripheral blood osteoclast precursors reside in the PM subpopulation, which is presumably a relatively less mature subpopulation and therefore possibly recently mobilized from bone marrow [19-21]. It has been proposed before [19-21,25] that, upon migration into inflammatory lesions, the PMs may contribute to the local macrophage proliferation which can be observed [40,41]. It is also possible that they could reside as osteoclast precursors in the synovial macrophage population within RA joints [30] which have been shown capable of differentiation into osteoclasts [42,43].

## Abbreviations

α-MEM: alpha-minimum essential medium; Cath K: cathepsin K; CFSE: carboxyfluorescein diacetate-succinimidyl ester; CTR: calcitonin receptor; FBS: fetal bovine serum; Hi-FBS: heat-inactivated fetal bovine serum; M-CSF: macrophage colony-stimulating factor; NP: non-proliferative; PBMC: peripheral blood mononuclear cell; PBS: phosphate-buffered saline; PCR: polymerase chain reaction; PM: proliferative monocyte; RA: rheumatoid arthritis; RANK: receptor activator of nuclear factor-kappa-B; RANKL: receptor activator of nuclear factor-kappa-B ligand; TRAP: tartrate-resistant acid phosphatase.

## Competing interests

The authors declare that they have no competing interests.

## Authors' contributions

RL designed and performed the study, analyzed the data, and drafted the manuscript. PDK performed the statistical analysis. JAH supervised the study and finalized the manuscript. All authors read and approved the final manuscript.
